# Direct estimation of regional lung volume change from paired and single CT images using residual regression neural network

**DOI:** 10.1002/mp.16365

**Published:** 2023-03-26

**Authors:** Sarah E. Gerard, Muhammad F. A. Chaudhary, Jacob Herrmann, Gary E. Christensen, Raúl San José Estépar, Joseph M. Reinhardt, Eric A. Hoffman

**Affiliations:** 1 Roy J. Carver Department of Biomedical Engineering, University of Iowa, Iowa City, Iowa, USA; 2 Department of Radiology, University of Iowa, Iowa City, Iowa, USA; 3 Department of Electrical and Computer Engineering, University of Iowa, Iowa City, Iowa, USA; 4 Department of Radiation Oncology, University of Iowa, Iowa City, Iowa, USA; 5 Department of Radiology, Harvard Medical School, Boston, Massachusetts, USA

**Keywords:** computed tomography, convolutional neural network, deep learning, image registration, Jacobian estimation, pulmonary imaging, pulmonary tissue mechanics

## Abstract

**Background:**

Chest computed tomography (CT) enables characterization of pulmonary diseases by producing high-resolution and high-contrast images of the intricate lung structures. Deformable image registration is used to align chest CT scans at different lung volumes, yielding estimates of local tissue expansion and contraction.

**Purpose:**

We investigated the utility of deep generative models for directly predicting local tissue volume change from lung CT images, bypassing computationally expensive iterative image registration and providing a method that can be utilized in scenarios where either one or two CT scans are available.

**Methods:**

A residual regression convolutional neural network, called Reg3DNet+, is proposed for directly regressing high-resolution images of local tissue volume change (i.e., Jacobian) from CT images. Image registration was performed between lung volumes at total lung capacity (TLC) and functional residual capacity (FRC) using a tissue mass- and structure-preserving registration algorithm. The Jacobian image was calculated from the registration-derived displacement field and used as the ground truth for local tissue volume change. Four separate Reg3DNet+ models were trained to predict Jacobian images using a multifactorial study design to compare the effects of network input (i.e., single image vs. paired images) and output space (i.e., FRC vs. TLC). The models were trained and evaluated on image datasets from the COPDGene study. Models were evaluated against the registration-derived Jacobian images using local, regional, and global evaluation metrics.

**Results:**

Statistical analysis revealed that both factors – network input and output space – were significant determinants for change in evaluation metrics. Paired-input models performed better than single-input models, and model performance was better in the output space of FRC rather than TLC. Mean structural similarity index for paired-input models was 0.959 and 0.956 for FRC and TLC output spaces, respectively, and for single-input models was 0.951 and 0.937. Global evaluation metrics demonstrated correlation between registration-derived Jacobian mean and predicted Jacobian mean: coefficient of determination (*r*^2^) for paired-input models was 0.974 and 0.938 for FRC and TLC output spaces, respectively, and for single-input models was 0.598 and 0.346. After correcting for effort, registration-derived lobar volume change was strongly correlated with the predicted lobar volume change:for paired-input models *r*^2^ was 0.899 for both FRC and TLC output spaces, and for single-input models *r*^2^ was 0.803 and 0.862, respectively.

**Conclusions:**

Convolutional neural networks can be used to directly predict local tissue mechanics, eliminating the need for computationally expensive image registration. Networks that use paired CT images acquired at TLC and FRC allow for more accurate prediction of local tissue expansion compared to networks that use a single image. Networks that only require a single input image still show promising results, particularly after correcting for effort, and allow for local tissue expansion estimation in cases where multiple CT scans are not available. For single-input networks, the FRC image is more predictive of local tissue volume change compared to the TLC image.

## 1 | INTRODUCTION

Quantitative computed tomography (QCT) is becoming increasingly common for characterizing various pulmonary abnormalities including cancer, chronic obstructive pulmonary disease (COPD), asthma, and cystic fibrosis.^1–4^ Recent developments are increasingly improving high-resolution chest CT scans that reveal regional structural and functional abnormalities within the lungs, providing distinct disease sub-phenotypes.^4–7^ A limitation to structural roadmaps of tissue destruction and remodeling is that they offer limited insight into the disease etiology and they do not provide a linkage to lung function. Lungs with considerable structural changes may show little change in spirometric measures and vice versa, such as underlying endothelial dysfunction,^8^ abnormal mucous properties,^9^ or peripheral small airways disease.^10–16^

Deformable image registration (DIR) of CT scans obtained at two or more lung volumes (e.g., at expiratory and inspiratory breath holds) is one approach which has moved away from simple structural metrics and offers an appropriate alternative for estimating functional information based on changes in local tissue expansion and contraction.^17,18^ From image matching of inspiratory and expiratory lung images has come an awareness of the role played by functional small airways disease (fSAD) as a precursor to parenchymal destruction.^19,20^ The Jacobian determinant (*J*) of the registration displacement field quantifies local volume change, and has been validated against a well-known, noninvasive method for estimating regional lung ventilation: xenon-enhanced CT (Xe-CT).^17,18^ Registration-derived biomechanical measures have since gained widespread attention for understanding COPD.^21–24^ Bodduluri et al. demonstrated that biomechanical features (extracted from Jacobians) were better predictors of COPD severity than conventional texture- and intensity-based features.^21^ Local change in lung volume averaged over the lung field was shown to be in better agreement with spirometry and was able to capture lung function decline and parenchymal tissue destruction.^22^ Moreover, mean Jacobian was significantly correlated with several indices of lung function and patient health, including forced expiratory volume in 1 second (FEV_1_), extent of emphysema, and 6-minute walk distance.^23^ These studies indicate the effectiveness of registration-based local tissue expansion measures for assessing COPD development and progression.

Image registration frameworks may not be feasible for some patients. For most patients, in a clinical setting, expiratory CT scans are simply not acquired. In carefully controlled academic environments, there is little or no coaching to functional residual capacity (FRC) or residual volume (RV) and thus the results from image matching are difficult to interpret. When there is coaching, some patients are simply unable to follow the specific instructions. An additional impediment is the fact that for frequent longitudinal assessments, despite 10 fold reductions in radiation doses from CT,^25,26^ there is a reluctance to expose patients repetitively to CT levels of radiation. There are also image processing issues with image registration methods. State-of-the-art lung image registration methods incur high computational costs for processing large 3D datasets. Registration methods relying on iterative optimization and subject-specific hyperparameter tuning are time-consuming, and are not practical for large-cohort COPD studies. However, the limitations of lung CT acquisition and registration do not undermine their significant clinical contributions. Rather, they highlight a need for alternative approaches to estimate CT-based local tissue mechanics that are robust, fast, scalable, and viable as an alternative to direct image registration or for predictions of regional mechanics (such as air trapping derived from Jacobians) even when only a single scan is available. Such a method may allow for salvaging a lung study when one of a lung volume pair is obtained at an inadequate inspiratory or expiratory effort or is degraded for some other reason.

Recently, deep generative modeling has emerged as a scalable and useful artificial intelligence (AI) tool for various image-to-image translation tasks using convolutional neural networks (CNNs). Applications in natural images include image denoising,^27^ super-resolution,^28^ style transfer,^29^ and data augmentation.^30^ Generative modeling is not limited to natural images, and has been successfully used for various medical image analysis tasks including image denoising,^31–34^ super-resolution,^35–37^ synthesis,^38–41^ segmentation,^42–45^ registration,^46–48^ and data augmentation.^49^ The success of deep generative modeling in other medical image-to-image translation tasks suggests its potential for CT-to-Jacobian prediction.

The typical registration-based approach to estimating local tissue expansion relies on aligning structures in two images acquired at different lung volumes. However, deep generative modeling may enable prediction of local tissue distensibility directly from a single CT image,^50^ instead relying on empirical structure-function relationships that link local intensity and texture to distensibility. For example, increased tissue density due to pulmonary edema or fibrosis may be associated with altered mechanical stiffness and inflation dynamics.^51,52^ Based on these structure–function relationships, we hypothesized that AI predictions of local tissue expansion using only a single lung CT image will be correlated with registration-derived Jacobian estimations, albeit to a lesser degree compared with AI predictions using paired images. Secondly, we hypothesize that single-image AI predictions will be more accurate using FRC images compared to total lung capacity (TLC) images, since FRC reveals pathophysiologic information not apparent at TLC, such as gas trapping.^53–55^ While several deep learning-based registration methods have been proposed which involve predicting the lung motion in three dimensions,^48^ predicting the Jacobian image directly only involves estimating the tissue distensibility without having to estimate motion making it a less complex task.

In our previous work, we demonstrated the feasibility of directly predicting a Jacobian image from paired CT images acquired at TLC and FRC using a convolutional neural network.^56^ In a separate study, we demonstrated the feasibility of directly predicting a Jacobian image from a single CT image acquired at RV using a generative adversarial network.^50^ The generative model in the latter study was trained on two-dimensional slices and was not guaranteed to be spatially consistent between adjacent slices. Additionally, these studies used different lung volumes for predicting Jacobian images and thus do not provide a consistent comparison between single- and paired-input Jacobian prediction methods. In this study, we performed a multifactorial design to assess Jacobian prediction by networks trained with either paired or single CT images, in each case estimating either local expansion from FRC or local contraction from TLC. There are three novel contributions in this work: (1) We provide a direct and fair comparison between single- and paired-input approaches for Jacobian prediction. The previous works were performed on different datasets, different lung volumes, different dimensionality, and different networks.(2) This is the first work to use TLC images for single-input models and compare it directly to using FRC for single-input models. The previous work using single-input models only used RV images as input.^50^ (3) For paired-input models, we report the combined effect of registration direction and prediction direction, that is, expansion from FRC versus contraction from TLC. The previous work with a paired-input model only reported prediction of expansion from FRC, using only registrations with moving image at TLC and fixed image at FRC.^56^

## 2 | METHODS

### 2.1 | Dataset

Chest CT image datasets from the COPDGene study were utilized in this study.^57^ COPDGene is a multicenter study that aims to identify genetic factors associated with COPD and characterize chest CT phenotypes of COPD.^57^ Each participant enrolled in COPDGene received two chest CT scans acquired at two lung volumes: TLC using 200 mAs, and FRC using 50 mAs. The images reconstructed from these scans will be referred to as *I*_TLC_ and *I*_FRC_. Disease severity was defined by the Global Initiative for Chronic Obstructive Lung Disease (GOLD), where GOLD 1 to 4 indicate mild to severe COPD and GOLD 0 includes asymptomatic smokers.^58^ For this study, 700 subjects were randomly sampled such that there was a uniform distribution of patients from each of the seven groups: the five GOLD levels, nonsmokers, and PRISm (preserved ratio impaired spirometry).^59^ The dataset was split into independent training and testing sets of 630 and 70 subjects, respectively, with uniform distribution of the seven groups.

### 2.2 | Image registration

Deformable image registration (DIR) estimates a transformation that describes the pointwise correspondence between two images: a fixed image *I*_F_ and a moving image *I*_M_. The transformation *h*(*x*_F_) maps points *x*_F_ from the fixed image space to their corresponding locations in the moving image space. The determinant of the Jacobian matrix of the transformation (henceforth referred to as simply the Jacobian or *J*) can be computed as a scalar-valued spatial map in the same spatial reference frame as the fixed image, that is, *J*_F_(*x*_F_) or more simply *J*_F_. The Jacobian thus describes the voxelwise multiplicative factor by which the local volume changes when the fixed image deforms to align with the moving image. *J*_F_(*x*_F_) > 1 indicates expansion at *x*_F_ and *J*_F_(*x*_F_) < 1 indicates contraction at *x*_F_.

**FIGURE 1:**
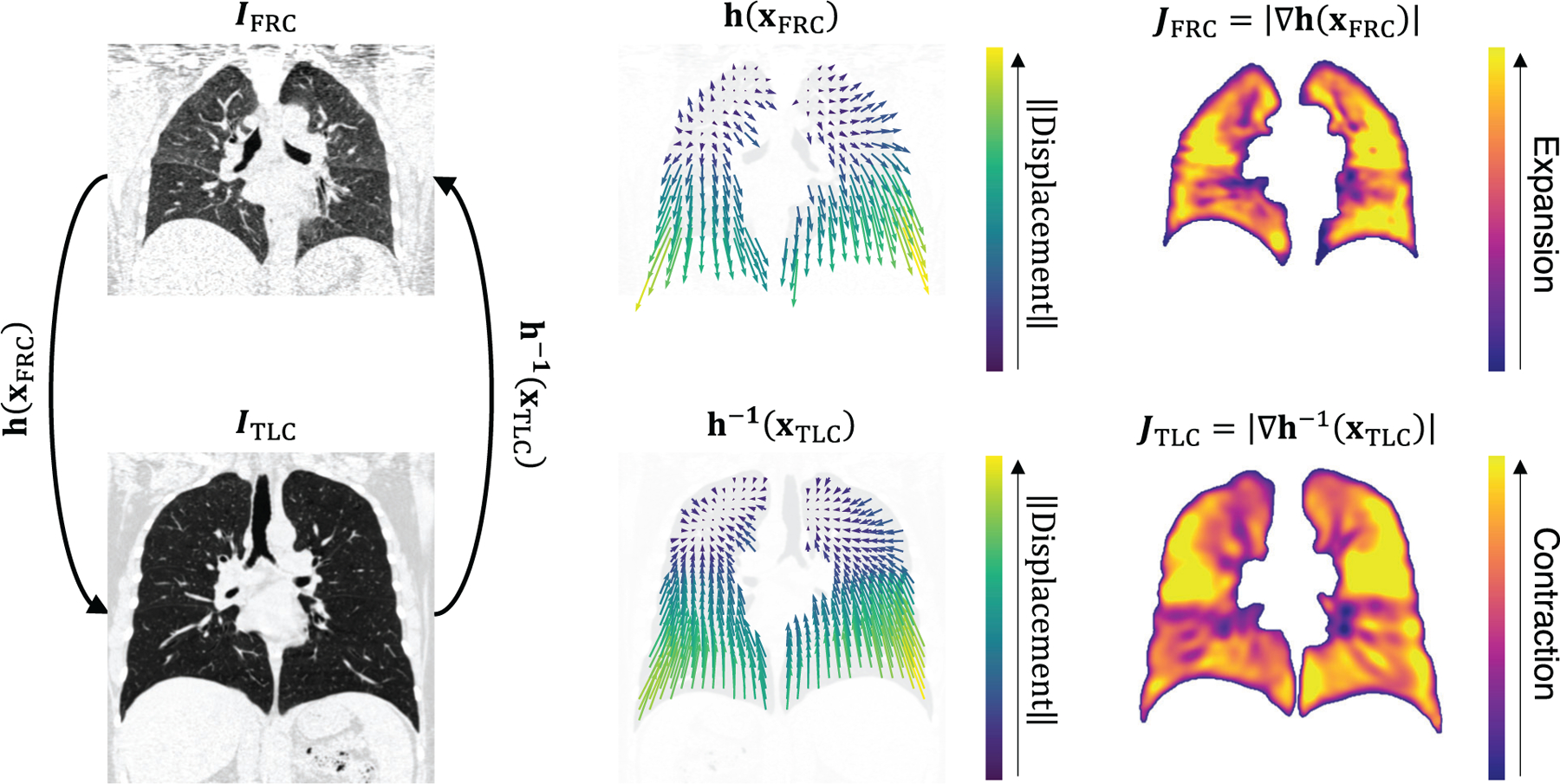
Image registration between two CT images acquired at different lung volumes, I_FRC_ and I_TLC_, is used to estimate a displacement vector field representing the anatomical correspondence between images (left column). The displacement vector field and Jacobian image are defined in the space of I_F_. Two different displacement vector fields (middle column) and Jacobian images (right column) can be estimated by performing the registration in both directions, that is, by alternating which image is defined as I_F_. If I_F_ = I_FRC_, h(x_FRC_) represents the mapping from points in IFRC to points in I_TLC_ and J_FRC_ represents the expansion from I_FRC_ to I_TLC_ (top middle and top right, respectively). By contrast, if I_F_ = I_TLC_ then h^−1^(x_TLC_) represents the mapping from points in I_TLC_ to points in I_FRC_ and J_TLC_ represents the contraction from I_TLC_ to I_FRC_ (bottom middle and bottom right, respectively).

Image registration can be performed in two directions between CT images acquired at FRC and TLC (Figure 1). The forward transformation *h*(*x*_FRC_) describes the deformation of moving image *I*_TLC_ to align with fixed image *I*_FRC_. Conversely, the inverse transformation *h*^−1^(*x*_TLC_) describes the deformation of moving image *I*_FRC_ to align with fixed image *I*_TLC_. The registration outputs *h*(*x*_F_) and *J*_F_ are always defined in the fixed image space (i.e., *I*_F_), and *h*(*x*_F_) is used to warp the moving image *I*_M_ to align with *I*_F_. Due to the opposing directions of the transformations, *J*_FRC_ represents expansion from *I*_FRC_ to *I*_TLC_, whereas *J*_TLC_ represents contraction from *I*_TLC_ to *I*_FRC_. For each subject in our dataset both *h*(*x*_FRC_) and *h*^−1^(*x*_TLC_) were estimated using a B-spline parameterized pyramid image registration algorithm.^17,18^ A tissue-mass-preserving cost function was utilized to account for changes in CT intensity at different lung volumes. Additionally, a structure term was added to the cost function to penalize misalignment of fissures and vessels. Fissure and vessel segmentations were obtained using FissureNet and a CNN for vessel segmentation.^60^ Prior to registration, *I*_FRC_ and *I*_TLC_ were masked using lung segmentations *M*_FRC_ and *M*_TLC_ obtained using a multi-resolution CNN.^61,62^ The registration-derived *J*_FRC_ and *J*_TLC_ images were used as ground truth for training CNNs for direct prediction of local tissue expansion.

### 2.3 | CNN Jacobian regression

#### 2.3.1 | Preprocessing

The following preprocessing was performed on all images for each subject: *I*_TLC_, *I*_FRC_, *J*_TLC_, *J*_FRC_. Gaussian smoothing was applied to prevent aliasing and then images were downsampled to 3 mm isotropic voxels. Images were cropped to the bounding box of *M*_TLC_ ∪ *M*_FRC_. FRC and TLC images were then masked using the corresponding mask downsampled with nearest-neighbor interpolation. Lastly, CT intensity values in *I*_TLC_ and *I*_FRC_ were clamped to the interval [−1024 HU, 200 HU] and then linearly rescaled to [−1, 1].

#### 2.3.2 | Reg3DNet+

**FIGURE 2:**
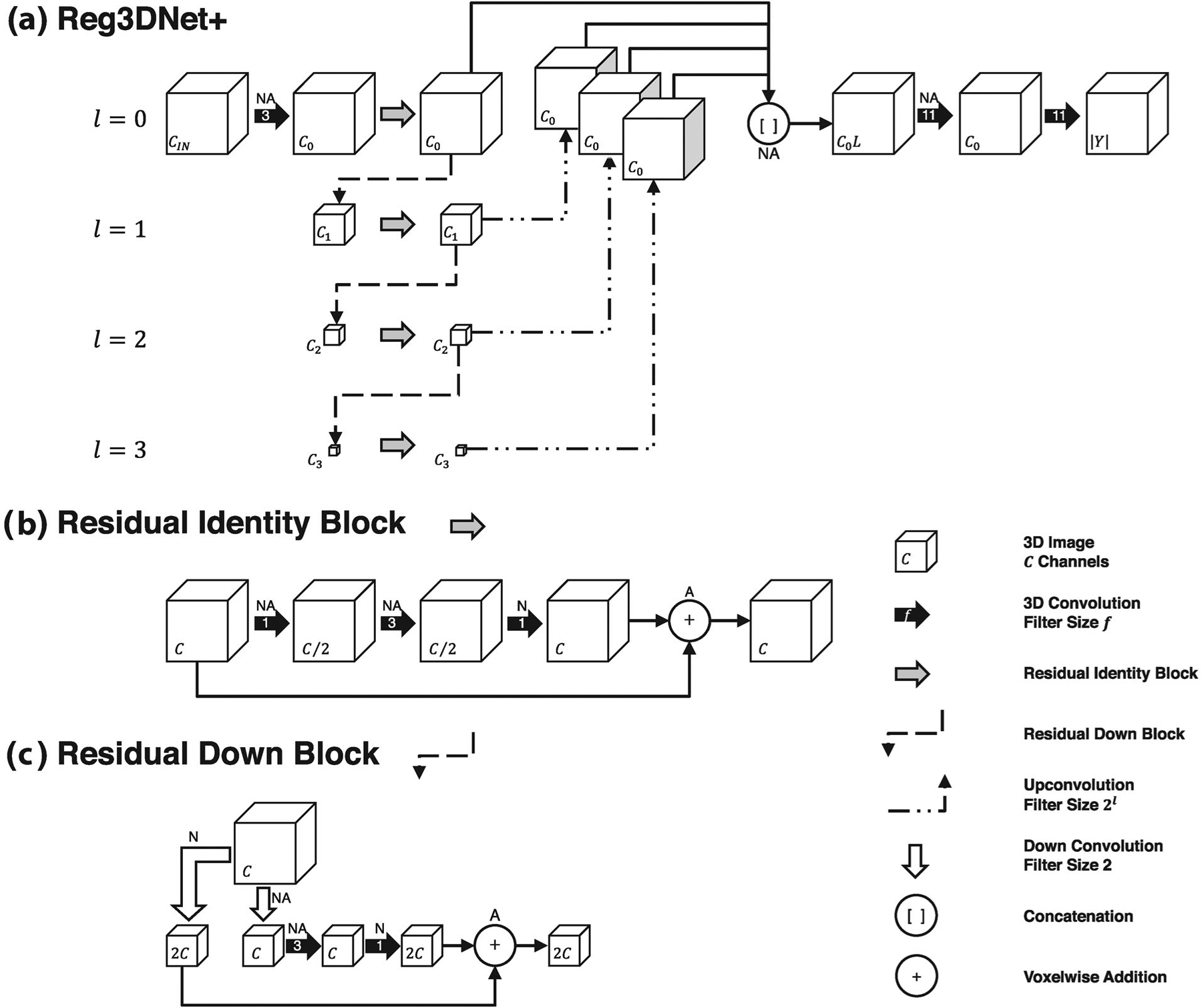
The overall Reg3DNet+ architecture proposed in this work is illustrated in panel a. The network consists of levels *l* = 0 … *L* - 1 which operate at different spatial resolutions. Each level consists of a residual identity block (gray arrow) followed by a residual down block (dashed arrow). The residual identity and residual down blocks are expanded in panel b and c, respectively. The output of the residual identity block from each level is upsampled to the original resolution through an upconvolution operation and the results are concatenated to create a multiscale representation. Lastly, two more convolutions are performed to generate the output image. Each cube represents a 3D image with the number of feature channels denoted in the lower left corner. A “N” and/or “A” above an operation denotes instance normalization and/or ReLU activation is performed following the operation.

In this work, we propose a deep learning approach for directly estimating *J* from single or paired CT images. The goal is to learn a model ${𝒢}_{\theta }$ with learnable parameters *θ* that maps a multidimensional input CT image to an output Jacobian image: ${𝒢}_{\theta }:I\to \hat{J}$ In the case of paired-image input, *I* in this expression is considered to be a four-dimensional image formed by the concatenation of the two three-dimensional CT images. The model ${𝒢}_{\theta }$ is parameterized by a CNN, which is a specialized neural network for exploiting spatial correlations in image data. We propose Reg3DNet+, which is a modified version of Seg3DNet – a memory efficient 3D fully convolutional encoder-decoder network (shown in Figure 2).^60^ Compared to Seg3DNet, Reg3DNet+ is a regression network that utilizes residual connections, or skip connections (+), which improves training in deep networks by allowing gradients to flow to earlier layers and thereby avoids vanishing gradients.

The input to Reg3DNet+ is *I*_TLC_ and/or *I*_FRC_. If both images are used, they are concatenated along a channel dimension, analogous to the channel dimension in an RGB image, and the number of channels is *C*_in_ = 2. The output of the network *Ŷ* is a single-channel image that is the same spatial size as the input image. Ground truth is defined as *Y* = log_2_ *J* such that reciprocal volume ratios have equal magnitudes and opposite signs (i.e., expansion at *x*: *Y*(*x*) > 0; contraction at *x*: *Y*(*x*) < 0). The final Jacobian prediction is recovered as $\hat{J}=2^{\hat{Y}}$ Predicting $\hat{J}$ is a regression task where each voxel in the target image takes on a continuous value, as opposed to a classification task where each voxel in the target image takes on a discrete value. Therefore, the output image has only one channel. Reg3DNet+ transforms the input CT images to the output *Ŷ* through a hierarchy of convolutional layers.

Reg3DNet+ consists of levels *l* = 0 … *L* − 1 which operate at different spatial resolutions. Each level consists of a residual identity block (Figure 2B) followed by a residual down block (Figure 2C). Residual identity blocks maintain both the spatial size and number of channels. Residual down blocks decrease the size of the feature maps by a factor of 2 along each dimension and increase the number of channels by a factor of 2. Both residual identity blocks and residual down blocks incorporate a residual connection allowing the identity function to be learned. The number of filters input to each residual identity block was *C*_*l*_ = 8 × 2^*l*^. The output of each residual identity block is upsampled to the original size using upconvolution with *C*_0_ filters, and the resulting maps are concatenated to create a multiscale feature representation of *C*_0_*L* channels. Lastly, two more convolutional layers are used to integrate the multi-scale features and produce *Ŷ*. All convolutional layers are followed by instance normalization and ReLU activation, except for the last layer. In residual blocks the normalization is performed before the residual connection and the activation is performed after the residual connection. In this work we use *L* = 4.

#### 2.3.3 | Training

To learn the model 𝒢_*θ*_ the parameters *θ* are optimized by minimizing a loss function which defines the dissimilarity between the ground truth *Y* and the network prediction *Ŷ*. A linear combination of the mean absolute error (MAE) and the structural similarity index (SSIM) was used as the loss function for training our model. The MAE loss between ground truth *Y* and network prediction *Ŷ* is defined as:

(1) $${ℒ}_{\text{MAE}}(Y,\hat{Y})=\frac{1}{\left|M_{\text{F}}\right|}\sum_{x\in M_{\text{F}}}\left|Y(x)-\hat{Y}(x)\right|$$

where *M*_F_ is the set of voxels in the lung mask for the *F* output space. The SSIM of two image patches *a* and *b* is:

(2) $$\text{SSIM(}a\text{, }b\text{)=}\frac{\left(2\mu_{a}\mu_{b\;}+C_{1})(2\sigma_{ab}+C_{2})\right)}{\left(\mu_{a}^{2}+\mu_{b}^{2}+C_{1})(\sigma_{a}^{2}+\sigma_{b}^{2}+C_{2}\right)}$$

where *μ*_*a*_ and *μ*_*b*_ are the Gaussian weighted mean of patch *a* and patch *b*, respectively, *σ*_*a*_ and *σ*_*b*_ are the Gaussian weighted standard deviation of patch *a* and patch *b*, respectively, and *σ*_*ab*_ is the Gaussian weighted covariance of patch *a* and patch *b*. *C*_1_ = (*K*_1_Δ)^2^ and *C*_2_ = (*K*_2_Δ)^2^ where Δ is the dynamic range of the image and *K*_1_ and *K*_2_ are constants. In this work we used an image patch of size 7^3^ voxels, a Gaussian weighting with *σ* = 1.5, Δ = 5, *K*_1_ = 0.01, and *K*_2_ = 0.03. The SSIM loss between the ground truth image *Y* and predicted image *Ŷ* is:

(3) $${ℒ}_{\text{SSIM}}(Y,\hat{Y})=\frac{1}{\left|M_{\text{F}}\right|}\sum_{x\in M_{\text{F}}}1-\text{SSIM}(w(Y(x)),w(\hat{Y}(x)))$$

where *w*(*Y*(*x*)) is a patch extracted from image *Y* centered at location *x*. The total loss function is defined as follows:

(4) $${ℒ}_{\text{total}}(Y,\hat{Y})=\alpha {ℒ}_{\text{MAE}}(Y,\hat{Y})+(1-\alpha ){ℒ}_{\text{SSIM}}(Y,\hat{Y})$$

In this work we use *α* = 0.5. Adam optimization was used with a learning rate of 1 × 10^−4^;^63^ the AMS-Grad variant of the algorithm was used to improve convergence.^64^ Parameters were initialized using He normal initialization.^65^

#### 2.3.4 | Implementation

TensorFlow was used to implement the deep learning models. The BioData Catalyst ecosystem^66^ was used for training and analysis. Specifically, NVIDIA V100 graphics cards with 32 GB RAM were used. To improve training efficiency and increase batchsize a multi-GPU training strategy was employed with 8 GPUs. A batchsize of 8 was used for each GPU, yielding an effective batchsize of 64. All models were trained for 1000 epochs, where each epoch took approximately 8 s. Compared to training with a single GPU, the multi-GPU setting on BioData Catalyst decreased training time by five-fold.

### 2.4 | Multifactorial design

**FIGURE 3:**
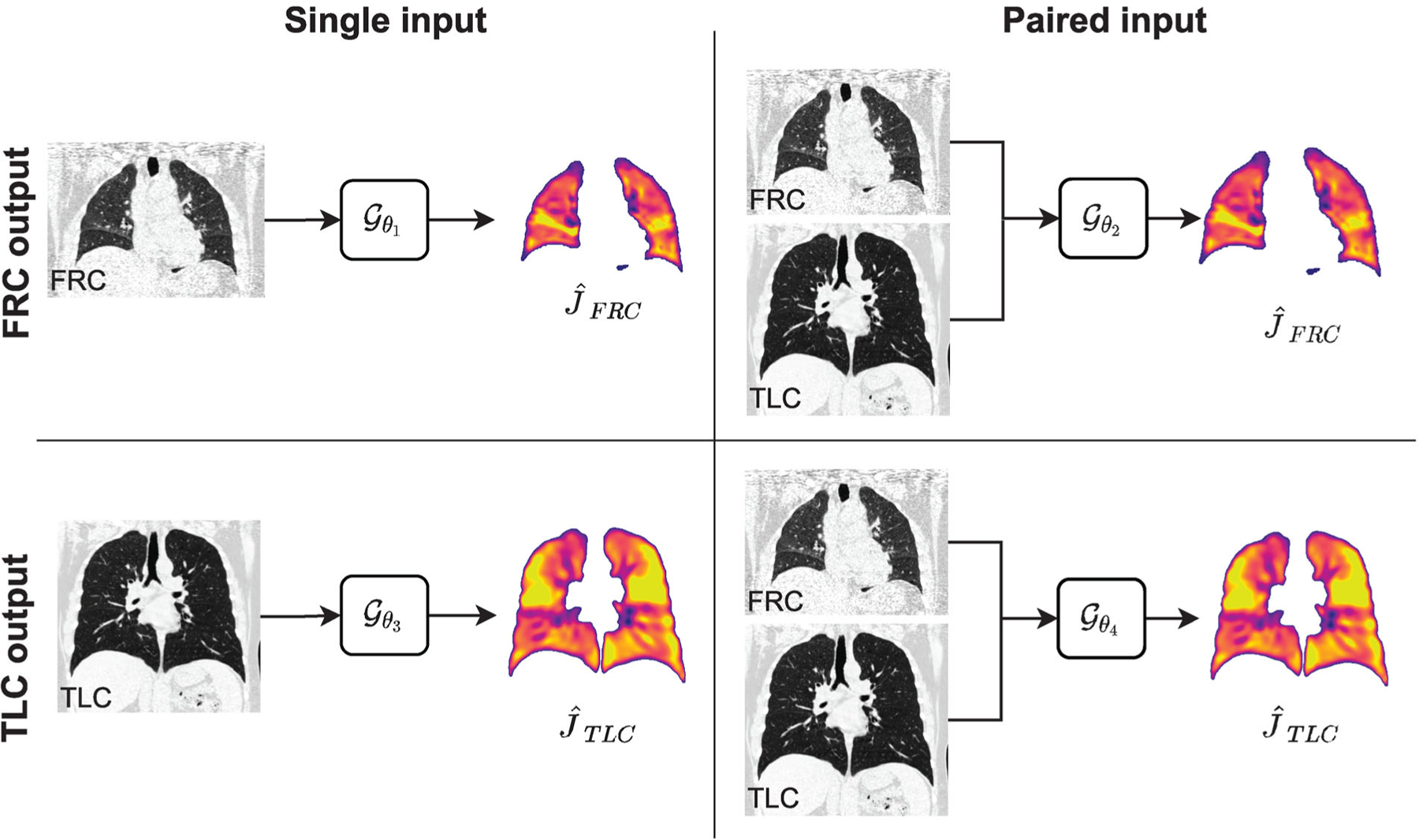
Multifactorial study design. Four Reg3DNet+ models were trained to study two factors: network input (columns) and output space (rows). The network input can be a single CT image (left column) or paired CT images (right column). The output space can be FRC (top row) or TLC (bottom row).

A multifactorial study was used to investigate two factors: network input and output space (Figure 3). Two levels were defined for each factor: single versus paired network input and FRC versus TLC output space. Note, the single-input networks only used the input image that matched the target output space, that is, *I*_FRC_ for the FRC output space (Figure 3 upper left quadrant) and *I*_TLC_ for the TLC output space (Figure 3 lower left quadrant).

### 2.5 | Evaluation

Each model was evaluated by quantitatively comparing the similarity of *Ĵ* and *J* images at local, regional, and global scales. At the local scale, voxelwise Spearman correlation, peak signal-to-noise ratio (PSNR), and MAE were computed. At the regional scale, SSIM and Dice coefficient were computed. The Dice coefficient was computed for high- and low-Jacobian lung regions which were defined for each patient by the upper and lower quartiles of Jacobian values, respectively. An aligned rank transform (ART) two-way repeated measures analysis of variance (ANOVA) was performed to analyze the main effects and interactions of the two factors in the multifactorial ablation study: network input and output space. This test was performed for all local and regional evaluation metrics.

At the global scale, the Jacobian mean (*J*_*μ*_) and coefficient of variation (*J*_*CV*_) were computed for the entire lung mask. Furthermore, we compared the distribution of Jacobian across different regions of interest (ROIs) by computing the volume change in each ROI (Δ*V*_ROI_) expressed as a percent of total lung volume change (Δ*V*_L_):

(5) $$\Delta V_{\text{ROI\%}}=100\frac{\Delta V_{\text{ROI}}}{\Delta V_{\text{L}}}$$

where:

(6) $$\Delta V_{\text{ROI}}=\left(J_{\mu ,\text{ROI}}-1\right)\cdot V_{\text{ROI}}$$

and where *J*_*μ*,ROI_ is the mean Jacobian in the ROI and *V*_ROI_ is the volume of the ROI. Two approaches were considered for partitioning the lung into non-overlapping ROIs. In the first approach, each lobe was considered a ROI, with lobar segmentations generated by a multiresolution CNN.^67^ In the second approach, ROIs were defined according to voxelwise classification as either normal, functional small airway disease (fSAD), or emphysema via parametric response mapping (PRM).^68^ Thresholds for PRM were −856 HU at FRC and −950 HU at TLC. For both the lobe- and PRM-defined ROIs, predicted Δ*V*_ROI_% was computed for each of the four models and compared to DIR Δ*V*_ROI_%.

## 3 | RESULTS

**FIGURE 4:**
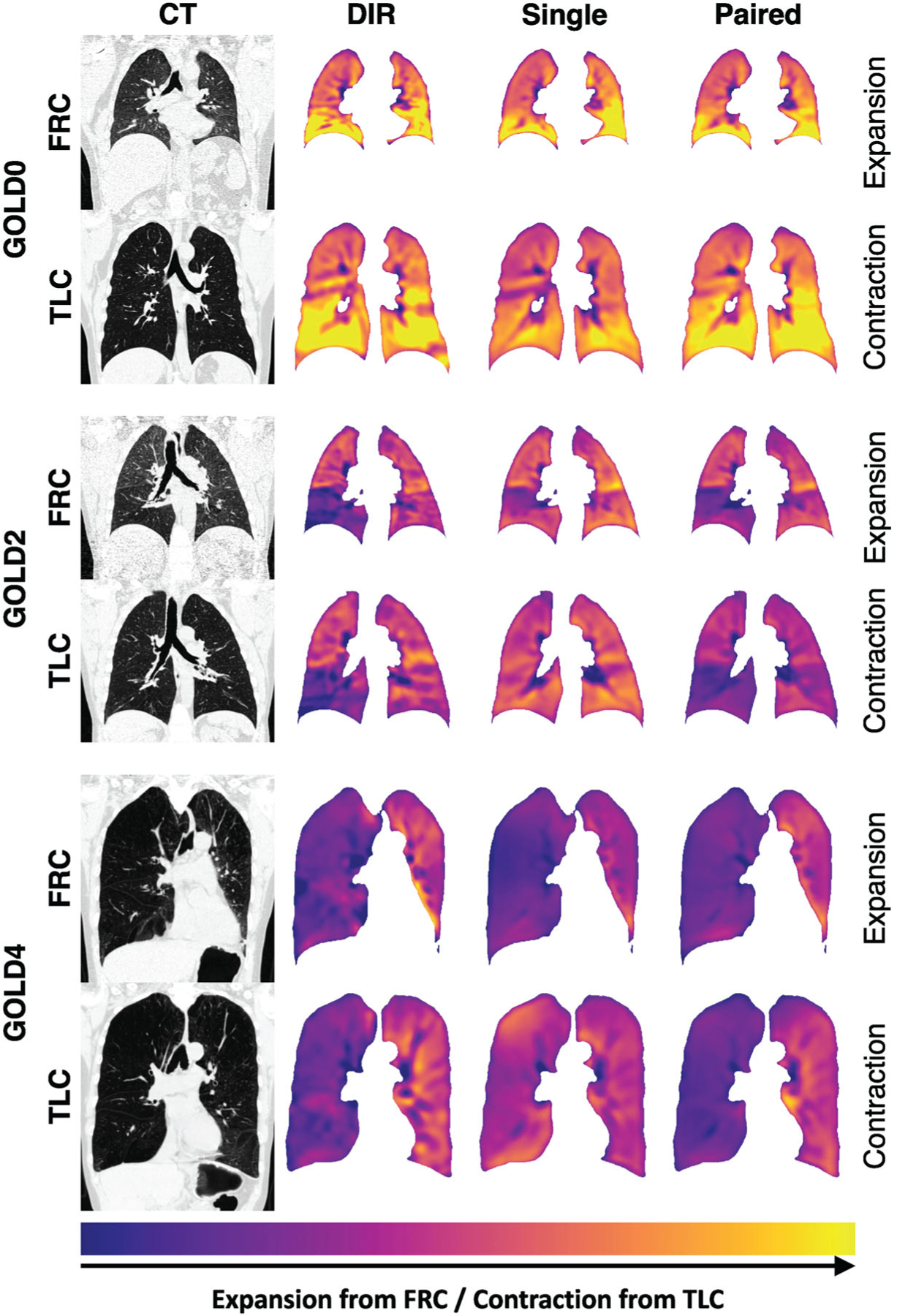
Qualitative results showing CT images and Jacobians on coronal slices. Three subjects are displayed, two rows for each subject corresponding to images in the FRC space and images in the TLC space. Note the the FRC and TLC rows for each subject show the images in the original space, therefore, there is no pointwise correspondence and it is not possible to show the exact same lung region. However, for consistency we chose the coronal slice with the carina for both FRC and TLC images. From left to right the columns show CT image, DIR result, Jacobian prediction of single-input network, and Jacobian prediction of paired-input network. From top to bottom different subjects are shown with increasing GOLD level.

Qualitative results of three subjects with different GOLD levels are displayed in Figure 4. Jacobians are transformed as|log_2_(*J*)| so that expansion from FRC and corresponding contraction from TLC can be visualized on the same color scale. Qualitatively, all AI models showed promising performance and were able to produce images that have similar spatial patterns of expansion or contraction compared to registration-based results. Figure 4 shows qualitative results for three cases with different GOLD levels. The least severe disease case (GOLD0) shows normal pattern of mechanics with more tissue expansion in the lower lobes compared to the upper lobes. All models performed similar and were able to predict this pattern. The GOLD2 case shows less overall expansion with a large region of very low expansion in the lower right lobes. All models except the single input model in the TLC space were able to replicate this pattern. The most severe disease case shows the lowest overall expansion, with large asymmetry between the left and right lungs. All models except the single input model in the TLC space were able to predict this asymmetry. The qualitative results demonstrate that for abnormal cases the *I*_FRC_ provides more predictive features.

**FIGURE 5:**
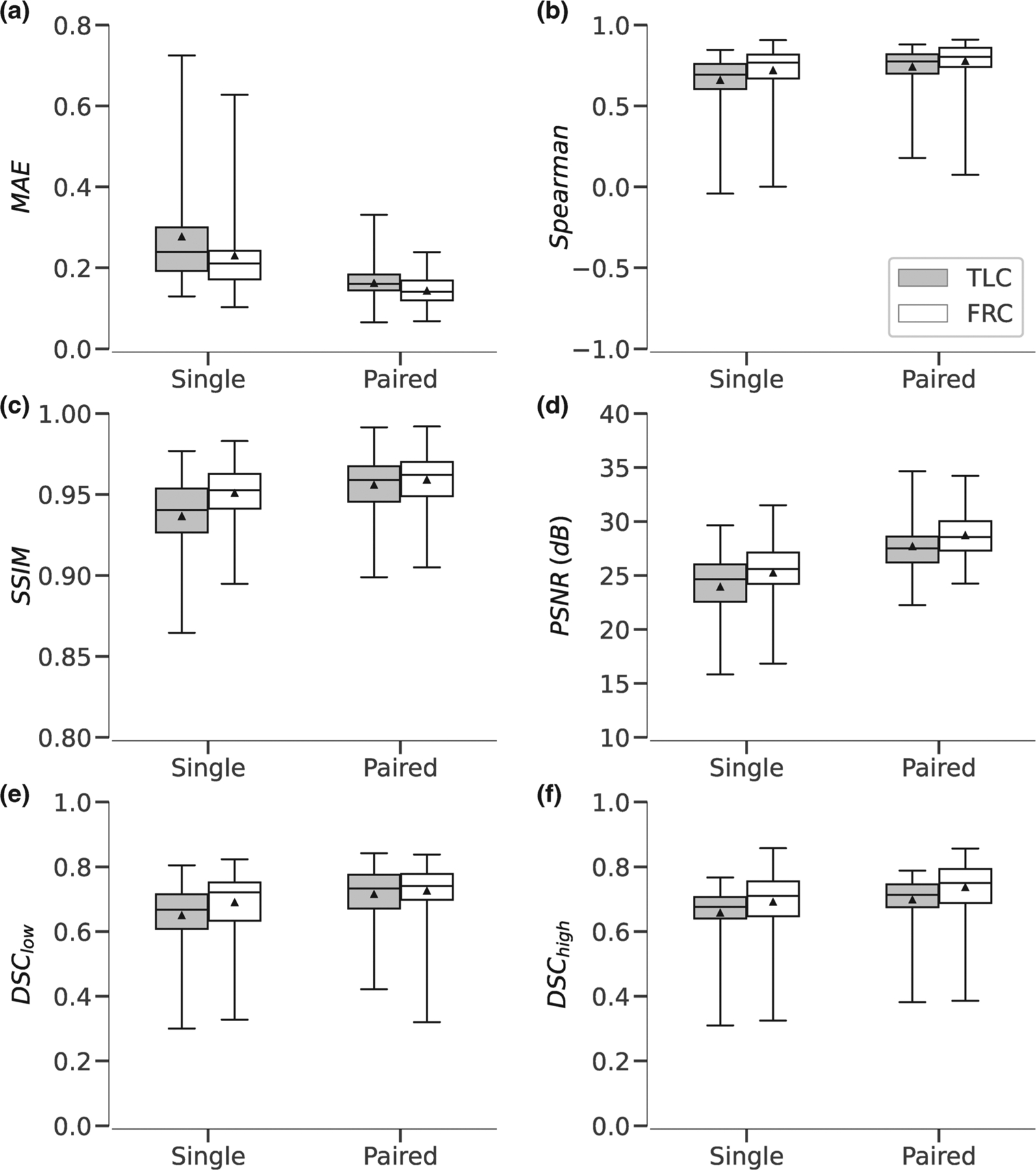
Local and regional quantitative evaluation metrics evaluated on testing dataset. Results are stratified by model. Each panel shows summary results for a different metric: (a) MAE, (b) Spearman correlation, (c) SSIM, (d) PSNR, (e) Dice low-Jacobian, and (f) Dice high-Jacobian. Note, a lower MAE is indicative of better performance whereas for the remaining metrics a higher value is indicative of better performance. Black triangles indicate mean values.

Box-plots for the local and regional quantitative evaluation metrics for each of the four models are displayed in Figure 5. The local and regional metrics were computed using |log_2_(*J*)| so that contraction and expansion ratios can be compared on the same scale. For all evaluation metrics, the ART ANOVA found both main effects, network input and output space, to be statistically significant factors (*p* < 1 × 10^−5^). For SSIM, Spearman, and Dice low-Jacobian, the interaction term was also statistically significant (*p* < 0.01).

**FIGURE 6:**
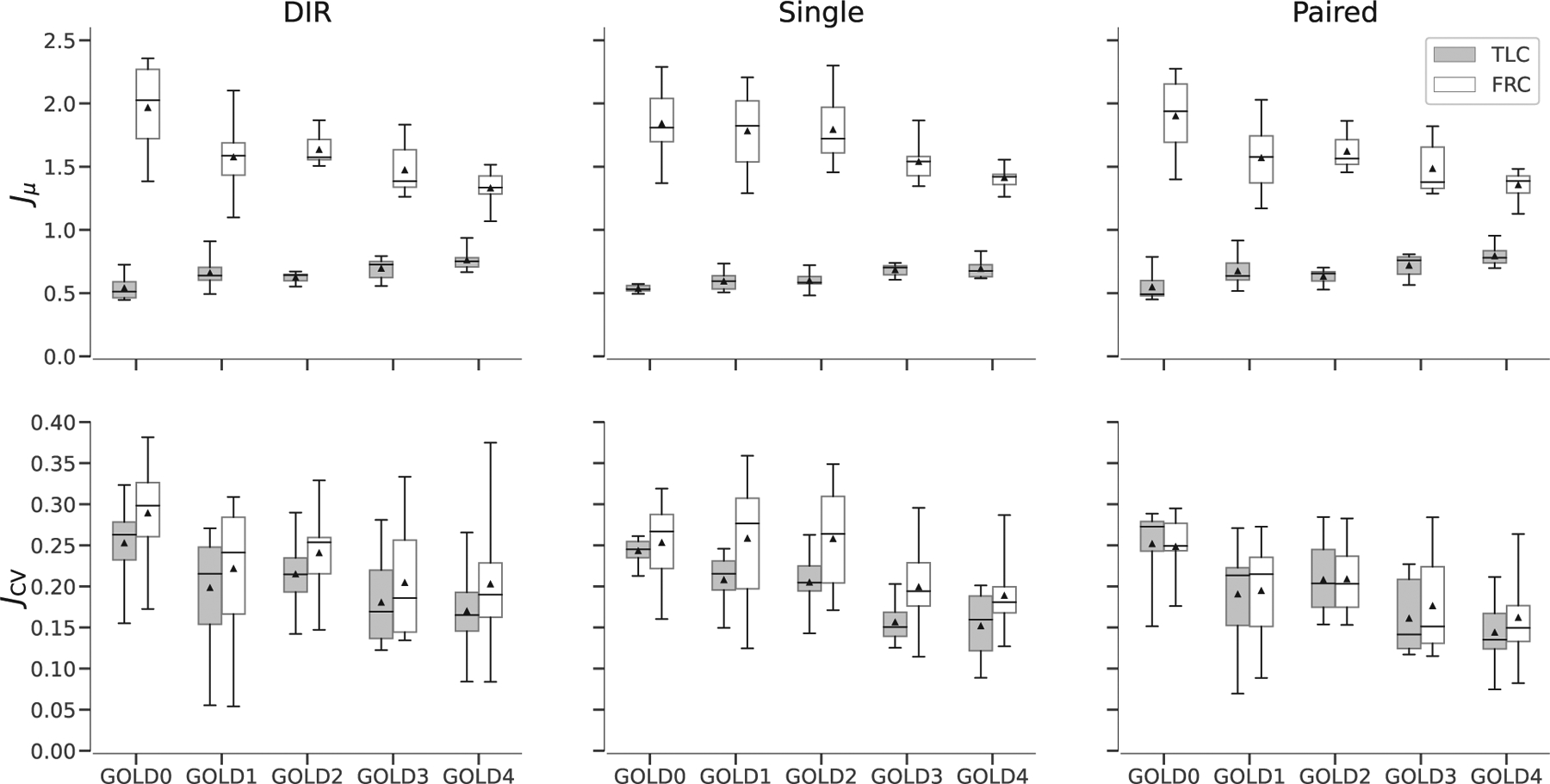
Global statistics of Jacobian images stratified by GOLD level. Top row shows trends in the *J*_*μ*_ and the bottom row shows trends in *J*_CV_. The columns from left to right show results for deformable image registration (DIR), single-input model, and paired-input model. Each panel shows results for prediction in the FRC output space (white) and TLC output space (gray).

Box-plots for global evaluation metrics are displayed in Figure 6. The global *J*_*μ*_ and *J*_CV_ were calculated within the lung field and the results were stratified by GOLD level. The plots show a decrease in *J*_*μ*_ with increasing GOLD level and a decrease in *J*_CV_ with increasing GOLD level. Note, a Jacobian of 1 indicates zero expansion or contraction; Jacobian greater than 1 indicates expansion whereas Jacobian less than 1 indicates contraction.

**FIGURE 7:**
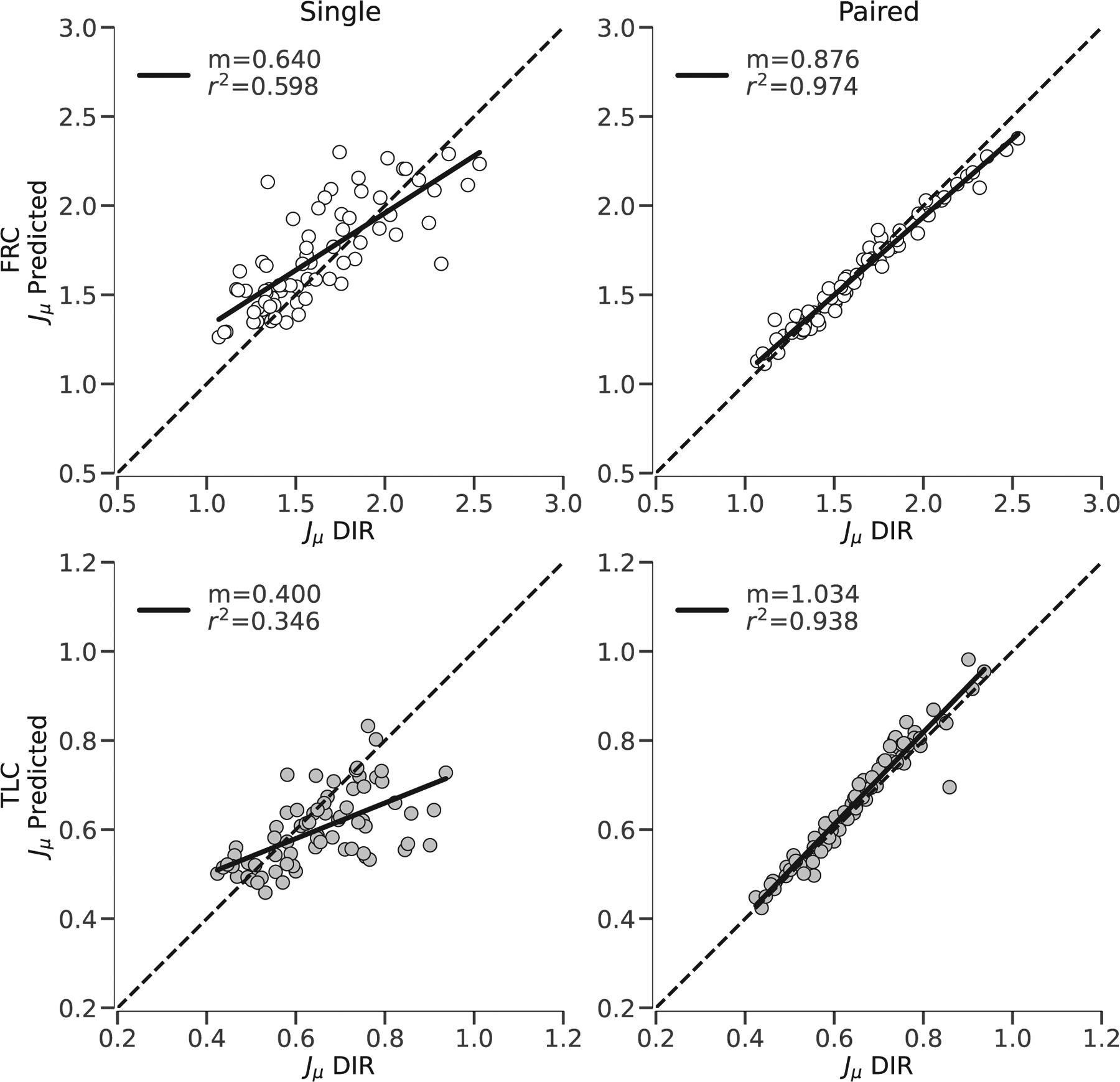
Scatter plots of *J*_*μ*_ for each subject, showing the value estimated by deformable image registration (DIR) compared to the value predicted by the four trained networks. Each panel corresponds to a different network, with rows corresponding to the output space (top: FRC, bottom: TLC), and columns corresponding to the network input (left: single image, right: paired images). Solid black line indicates fitted linear regression model. Dashed black line indicates line of identity for reference.

Figure 7 shows scatter plots of network-predicted versus registration-derived *J*_*μ*_ for each subject. A linear regression was computed for each combination of network input (single vs. paired) and output space (FRC vs. TLC) and the coefficient of determination *r*^2^ was computed. For paired-input models *r*^2^ = 0.974 and 0.938 for FRC and TLC, respectively, and for single-input models *r*^2^ = 0.598 and 0.346 for FRC and TLC, respectively. For paired-input models, note that although the FRC output space was associated with a higher *r*^2^, the TLC output space was associated with a regression slope closer to identity.

**FIGURE 8:**
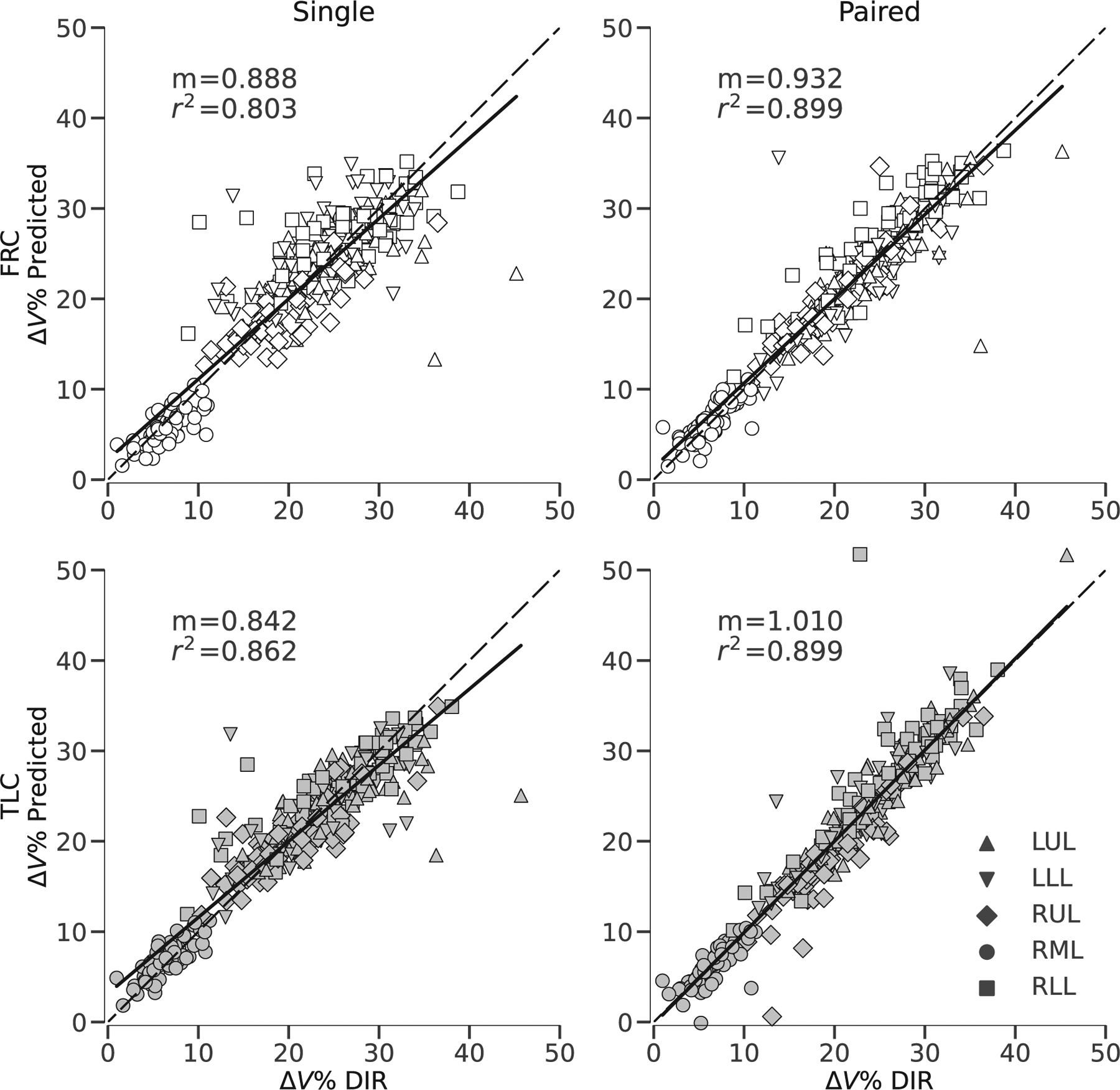
Scatter plots of the volume change by lobe region expressed as a percent of total lung volume change (Δ*V* %) for each subject, showing the value estimated by deformable image registration (DIR) compared to the value predicted by the four trained networks. Each panel corresponds to a different network, with rows corresponding to the output space (top: FRC, bottom: TLC), and columns corresponding to the network input (left: single image, right: paired images). Solid black line indicates fitted linear regression model. The different lobes are denoted with different symbols. Dashed black line indicates line of identity for reference.

**FIGURE 9:**
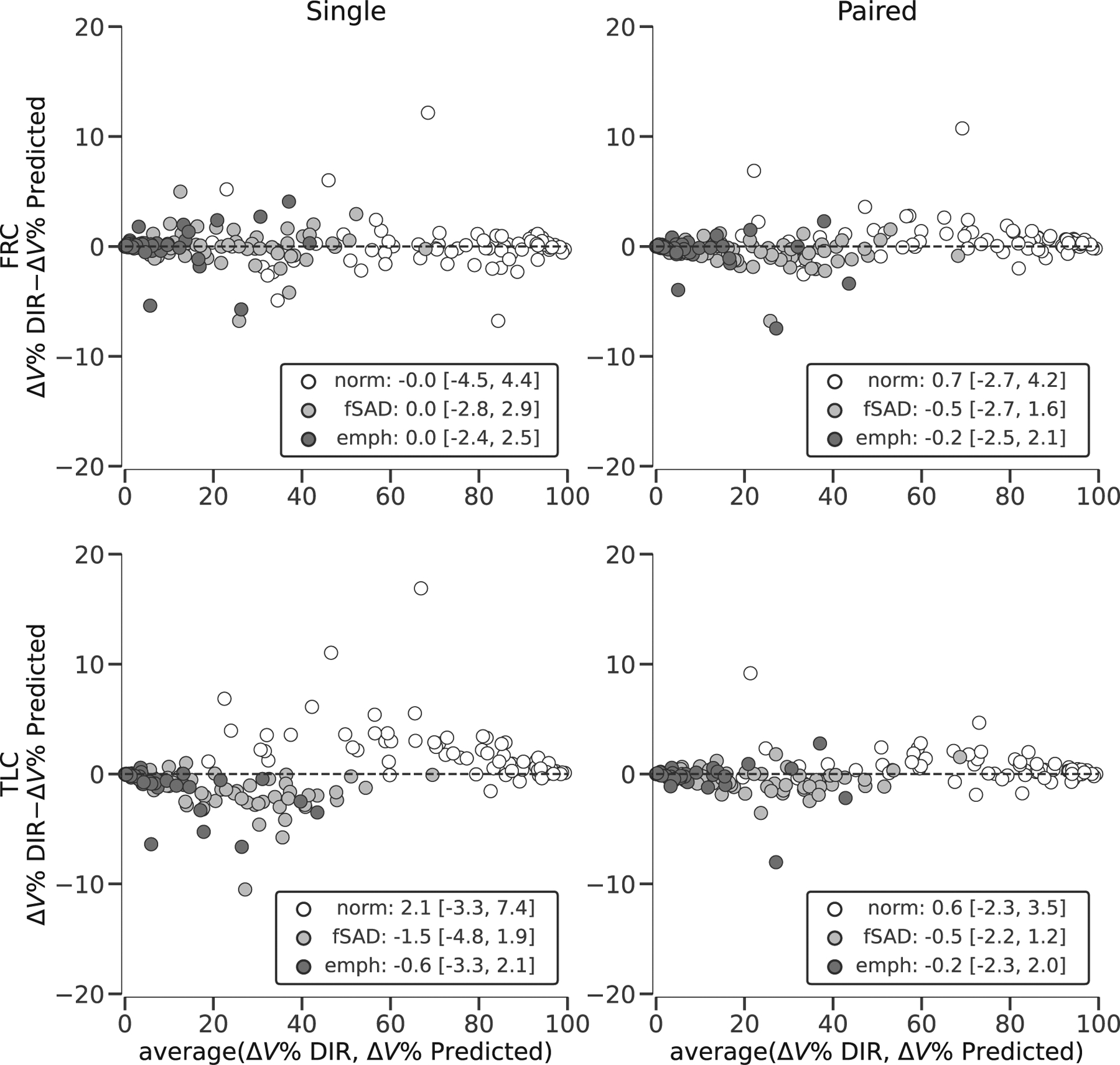
Bland-Altman plots for the volume change by PRM region expressed as a percent of total lung volume change (Δ*V* %) predicted by each of the four trained networks compared to the ground truth estimated by deformable image registration (DIR). Each panel corresponds to a different network, with rows corresponding to the output space (top: FRC, bottom: TLC), and columns corresponding to the network input (left: single image, right: paired images). The mean and 95% limits of agreement (mean ±1.96 standard deviation of the difference) for each PRM region are displayed in the legend. Dashed line is fixed at zero for reference.

Figure 8 shows scatter plots of network-predicted versus registration-derived Δ*V*_ROI_% using lobar ROIs. Linear regressions and coefficients of determination *r*^2^ were computed for each model. For paired-input models *r*^2^ = 0.899 for both FRC and TLC, and for single-input models *r*^2^ = 0.803 and 0.862 for FRC and TLC, respectively. Figure 9 shows Bland-Altman plots for Δ*V*_ROI_% using PRM-derived ROIs. The different PRM categories are denoted by color and the mean and 95% limits of agreement were computed for each category (i.e., normal, fSAD, and emphysema). To avoid divergence of Δ*V*_ROI_% calculation (i.e., division by near-zero values), subjects with almost no total lung volume change (|*J*_*μ*_ − 1| < 0.02) were excluded from this analysis. This resulted in one subject being excluded.

## 4 | DISCUSSION

In this work, a deep learning approach for local volume change prediction in CT images was proposed. Deformable image registration is the standard approach for estimating local volume change, but it is an iterative process than can take several hours especially for large volumetric medical images. The proposed AI framework enables Jacobian images to be directly predicted from single or paired CT images in less than a minute. This enables high-throughput processing and real-time analysis in clinical and research settings. Furthermore, the single-input models enable biomechanical assessment even when only a single CT image is available – an impossible task for image registration. In this study, deformable image registration was used to generate ground truth images in FRC and TLC output spaces – *J*_FRC_ and *J*_TLC_ – with network input being either a pair of CT images (*I*_FRC_ and *I*_TLC_) or only a single CT image (*I*_FRC_ or *I*_TLC_). The Reg3DNet+ architecture was proposed for directly predicting Jacobian images from CT images. A multifactorial study was designed to test the effects of network input and output space.

A dataset of CT scans in COPD patients was used for training and evaluation.^57^ Overall there was a decrease in global *J*_*μ*_ for patients with increasing GOLD level, and a decrease in global *J*_CV_. These findings are consistent with previous literature indicating that Jacobians are reliable biomarkers of COPD severity.^23,56,69^

The ART ANOVA found network input to be a significant factor for all local and regional evaluation metrics. As expected, models with paired-image input (*I*_TLC_ and *I*_FRC_) outperformed models with single-image input (*I*_TLC_ or *I*_FRC_), by 15% for PSNR and by 1.5% for SSIM. The benefit of using paired-image input is likely attributed to the availability of information about both inflation states simultaneously. We speculate that the paired-image network is able to learn convolutional filters that correlate distinct structural features and their corresponding deformation between the two input images. By contrast, predicting expansion and contraction based on only a single image is a difficult task, because it requires inference of mechanical properties and distensibility based on apparent CT intensity and texture alone. Nonetheless the single-input models exhibited high performance. The small 1.5% difference in SSIM (Figure 5) indicates that single-input models are able to predict spatial patterns with roughly the same degree of accuracy as the paired-image models. However, the larger 15% difference in PSNR (Figure 5) and 63% to 171% difference in global mean Jacobian *r*^2^ (Figure 7) indicates that the exact magnitude of volume change is less predictable in single-input models which is expected since magnitude is, in part, effort dependent.

The ART ANOVA also found output space to be a significant factor affecting Jacobian prediction. Specifically, models predicting Jacobian in the FRC output space performed better than models predicting Jacobian in the TLC output space. For several of the evaluation metrics (i.e., Spearman, SSIM, Dice low-Jacobian), the interaction term between network input and output space was significant. This indicates that the effect of output space is dependent on the network input, that is, there is a greater difference between metrics for TLC and FRC output space in single-input models than in paired-input models. Specifically, *I*_FRC_ provides more predictive information than *I*_TLC_ especially for single-input models. The *r*^2^ for global *J*_*μ*_ DIR vs. *J*_*μ*_ predicted regressions was also much higher for the single-input FRC output space (0.598) than for the single-input TLC output space (0.346).

After correcting for effort dependent variability by normalizing regional volume changes to total lung volume change, we found that network prediction for both lobar- and PRM-based ROIs were consistent with DIR estimates. The *r*^2^ for these lobe-based effort-corrected regressions all indicate a strong correlation (i.e., *r*^2^ >= 803) which when contrasted against the low *r*^2^ for global mean Jacobian for single-input models, indicates that regional heterogeneity of Jacobian prediction is accurate, despite weak ability to predict the effort dependent global mean Jacobian. We also repeated this analysis using PRM-defined ROIs, which also supports this notion and furthermore indicates that model predictions are sensitive to structural indications of pathological alterations, for example, emphysema and fSAD. We do note however that these structural indications (i.e., gas trapping and emphysema) are less obvious in TLC scans,^53,55^ which results in larger differences between DIR and predictions for single-input TLC model. This is illustrated in Figure 9 where compared to the other models, the single-input TLC model overestimated volume change in fSAD regions and underestimated volume change in normal regions. The addition of spirometry to inform the total volume change from FRC to TLC could augment the single-input models for a more accurate prediction of absolute volume changes rather than relative volume changes.

In this study, the performance of single-input models provides confidence that appearance in CT images (e.g., intensity, texture, location) is linked to tissue distensibility, such that inherent structure–function relationships may permit clinical assessment of inflation heterogeneity, spatial gradients, and regional defects using only a single CT image. Furthermore, it may be possible to combine single-image assessment together with global measurements of respiratory mechanics (e.g., spirometry, compliance) to accurately predict both the spatial patterns and the local magnitudes of volume change, without a second CT scan. Although it may be nonintuitive to use a single image for a task typically requiring two images, deep learning approaches benefit from their ability to discover abstract yet predictive features within single images directly from training data. Another application involving monocular depth perception (i.e., from single images as opposed to paired stereoscopic images) was also successfully performed by AI models, with attempts to decipher which image features convey predictive information by testing modified inputs.^70^ Relying on single-image models with CT scans at FRC–and forgoing a second CT scan at TLC–has benefits including reduced radiation exposure and reduced Jacobian variability associated with effort-dependent inspiration to TLC. The current study focused on a multicenter study of smoking associated COPD. For deep learning models to predict regional lung function from single or even pairs of lung images across a variety of clinical presentations, it is likely that a broader training set will be required.

Future studies will investigate whether images acquired at RV improve performance compared to FRC images for single-input models. RV is the lung volume used in pulmonary function laboratories to assess air trapping. FRC was the expiratory volume used in COPDGene because of historical reasons. When the study began, it was difficult for patients to hold their breath for the 10–20 s required for image acquisition. Current scan times can be well under 5 s. In addition, it may be worth investigating whether prediction performance is enhanced when the network input includes a combination of more than two images (e.g., RV, FRC, and TLC), compared to only a lung volume pair. It is unclear why there is a difference in performance associated with TLC versus FRC output spaces for paired-input models. It is possible that deformable image registration is less reliable when registering small lung volumes to large lung volumes (i.e., using TLC output space or *I*_TLC_ fixed image),^71,72^ and thus the registration-derived Jacobian used as ground truth is less predictable. It is also unclear whether image quality and signal-to-noise ratio influence model training or prediction, given that CT scans were performed with lower dose (i.e., higher noise) at FRC (50 mAs) compared to TLC (200 mAs). Whatever the reason may be for the observed differences in performance, the results presented herein suggest that AI-based assessment of regional lung volume change with only a single CT scan is more performant if the scan is acquired at FRC rather than TLC. In this work the single- and paired-input models were trained independently. An alternate approach would be to train one network that could accept either single or paired inputs using missing data approaches, which may improve the predictive power of the network.

A limitation of this work is that the registration-derived estimates used as ground truth for training AI models in this study are not necessarily error-free. The ground truth *J* images were derived from a deformable image registration algorithm which has demonstrated low registration error and high correlation with ventilation imaging modalities.^18,73,74^ However, image registration is an iterative process that is subject to errors due to convergence failures or convergence to local minima.^71^ Registration is especially difficult for complex, nonaffine, and large deformations which occur in the lung between FRC and TLC. Furthermore, B-spline parameterized registration can only describe smooth deformations, and thus cannot account for sliding that occurs between lobes and at the chest wall.^75^ Biases that exist in the registration algorithm may inadvertently be learned by the network.^76^ Another limitation of this work is the models were trained on downsampled images in order to train on full 3D volumes given constraints in GPU memory. While low-resolution images allows for more global and 3D context, there is a loss of small-scale structures present in high-resolution images that are likely predictive of tissue distensibility.

## 5 | CONCLUSION

In this work, we explored a deep learning approach to directly predicting regional lung volume change in CT images, bypassing the time-consuming process of image registration. Moreover, we investigated the potential for predicting regional lung volume change from a single CT image, bypassing the need for multiple CT scans and additional radiation exposure. Paired-input models predicting Jacobian images in the FRC output space demonstrated the best performance in a cohort of COPD patients. Nonetheless, single-input models showed promising performance, especially when used with images acquired at FRC rather than TLC. The single-input model regional performance was comparable to the paired-input after correction for effort, indicating that the regional heterogeneity of Jacobian single-input prediction is accurate, despite weak ability to predict the effort dependent total Jacobian mean. The multifactorial study design used in this work yielded novel insights into the presence of structure-function relationships between lung tissue appearance in CT images and regional distensibility. Such intrinsic relationships underlie the success of AI models in predicting volume change based on only a single image, which is a nonintuitive task for humans as well as an impossible task for image registration.
